# A similar injury profile observed in franchise men’s and women’s cricket in England and Wales: injury surveillance analysis from the first three ‘The Hundred’ competitions

**DOI:** 10.1136/bmjsem-2023-001815

**Published:** 2024-01-23

**Authors:** Amy Williams, Nicholas Peirce, Steve Griffin, Ben Langley, Anna Warren, Thamindu Wedatilake, Luke Goggins, Carly D McKay, Keith A Stokes, Sean Williams

**Affiliations:** 1 Department for Health, Centre for Health and Injury & Illness Prevention in Sport, University of Bath, Bath, UK; 2 UK Collaborating Centre on Injury and Illness Prevention in Sport (UKCCIIS), Edinburgh & Bath, UK; 3 Science and Medicine, England and Wales Cricket Board, Loughborough, UK; 4 Mumbai Indians, Mumbai, India

**Keywords:** Cricket, Sporting injuries, Epidemiology, Prevention

## Abstract

**Objectives:**

To describe the injury profile of a novel format cricket competition (‘The Hundred’) and compare injury incidence and prevalence between the men’s and women’s competitions.

**Methods:**

Medical staff prospectively collected injury data from the eight men’s and women’s teams during the 2021–2023 competitions. Injury definitions and incidence calculations followed the international consensus statement.

**Results:**

In the men’s competition, 164 injuries were recorded, compared with 127 in the women’s competition. Tournament injury incidence was 36.6 (95% CI 31.4 to 42.7) and 32.5 (95% CI 27.3 to 38.7)/100 players/tournament in the men’s and women’s competition, respectively. Non-time-loss incidence (men’s 26.6 (95% CI 22.2 to 31.8), women’s 24.6 (95% CI 20.1 to 30.0)/100 players/tournament) was higher than time-loss incidence (men’s 10.0 (95% CI 7.5 to 13.5), women’s 7.9 (95% CI 5.6 to 11.3)/100 players/tournament). Injury prevalence was 2.9% and 3.6% in the men’s and women’s competitions, respectively. Match fielding was the most common activity at injury in both competitions. The thigh and hand were the most common body location time-loss injury in the men’s and women’s competitions, respectively.

**Conclusion:**

A similar injury profile was observed between the men’s and women’s competition. Preventative strategies targeting thigh injuries in the men’s competition and hand injuries in the women’s competition would be beneficial. Compared with published injury rates, ‘The Hundred’ men’s presents a greater risk of injury than Twenty20 (T20), but similar to one-day cricket, with ‘The Hundred’ women’s presenting a similar injury risk to T20 and one-day cricket. Additional years of data are required to confirm these findings.

**WHAT IS ALREADY KNOWN ON THIS TOPIC**

- Limited-overs men’s cricket presents a greater risk of injury than in multiday cricket, with the injury rate in men’s one-day and Twenty20 (T20) cricket higher than in women’s T20 and one-day cricket.
- Understanding the risk of injury in a new competition and differences in injury profiles between men and women cricketers in a similar setting is required.
- The impact of franchise cricket in England and Wales on men and women cricket players is unknown.

**WHAT THIS STUDY ADDS**

- A similar tournament and match injury incidence was found between the men’s and women’s competitions.
- On any given day, 2.9% of players in the men’s ‘The Hundred’ and 3.6% of players in the women’s ‘The Hundred’ were unavailable due to injury.
- Match fielding was associated with the highest time-loss and non-time-loss injury incidence for both competitions.
- For men, ‘The Hundred’ presents a greater risk of injury than T20, but similar to one-day cricket, whereas for women, ‘The Hundred’ has a similar risk of injury to T20 and one-day men’s cricket in England and Wales.

**HOW THIS STUDY MIGHT AFFECT RESEARCH, PRACTICE OR POLICY**

- Information on the expected injury rates during ‘The Hundred’ is provided to inform men’s and women’s squad planning throughout the competition.
- Highlights fielding during matches as an injury prevention priority in short-format cricket for both men and women.
- Identifies the risk of thigh injuries in the men’s competition and hand and shoulder injuries in the women’s competition, where developing injury prevention strategies to reduce the risk would be beneficial.

## Introduction

Franchise Twenty20 (T20) cricket has grown worldwide to include men’s and women’s competitions. In 2021, the England and Wales Cricket Board (ECB) introduced a novel format of professional franchise cricket tournament, ‘The Hundred’, taking place over 4 weeks during the domestic cricket season. ‘The Hundred’ features eight men’s and eight women’s teams competing in respective round-robin style leagues with the same match scheduling. Traditional overs (6 balls) are replaced by a set of 5 deliveries (fives), with bowlers permitted to bowl 5 or 10 consecutive balls at once with a limit of 20 deliveries per game. Each inning consists of 100 balls, 20 less than T20, making it the shortest format of cricket in England and Wales.

Injury surveillance is deemed the first step towards injury prevention in athletes.^1^ One-day cricket (50 overs) has been consistently reported as the format with the highest injury incidence in men’s cricket.^2–4^ While there are fewer injury epidemiology studies in elite women’s cricket, comparison of injury profiles between elite men’s and women’s Australian cricket players has revealed a higher match injury incidence in men’s T20 and one-day cricket compared with women’s T20 and one-day cricket.^5^ Comparisons of injury rates between men’s and women’s domestic cricket are difficult due to differences in match scheduling and available rest days, potentially influencing injury risk.

‘The Hundred’ provides the first opportunity in elite cricket to directly compare men’s and women’s cricket injury profiles in the same format, with similar match scheduling and recovery periods. Understanding the risk of injury in a new competition will provide important insight that can be used to identify whether players are at an increased risk of injury compared with established competitions, ultimately informing injury prevention initiatives.^1^ This study aims to describe the injury profile of the ‘The Hundred’ and compare injury incidence and prevalence between the men’s and women’s competitions.

## Methods

### Participants

Data were collected from the first three ‘The Hundred’ competitions: 2021 (21 July–22 August), 2022 (3 August–3 September) and 2023 (1 August–27 August). All registered men’s and women’s players from eight franchises were included in the study.

### Procedures

All injuries were recorded by the teams’ medical staff on a purpose-built online database (Cricket Squad (The Sports Office, UK)) at the time of injury. Consistent with the consensus statement, the definition of injury included illness.^6^ All names were removed and replaced with a unique numerical ID by a gatekeeper at the ECB before data sharing with the University research partner. The data were checked by the lead author, who implemented a quality control process to check for errors and duplicates in the data.

Conforming with the updated consensus statement, two injury definitions were used,^6^ applied retrospectively.

1. Tournament injuries were defined as medical attention injuries where ‘any health-related condition that required medical (or medical staff) attention and had the potential to affect cricket training or playing’, that occurred during the tournament.^6^ This definition includes time-loss (TL) and non-TL (NTL) injuries. Only injuries sustained during ‘The Hundred’ were included. An injury was considered a TL if a player was considered unavailable for match-play, irrespective of whether a match or training was scheduled on the day(s) the player was unavailable. An injury was considered NTL if a player was considered available for match-play, irrespective of whether a match or training was scheduled on the days(s) of the injury. Annual incidence is recommended per 100 players per year,^6^ however, given the short duration of this tournament, it did not seem appropriate to extrapolate the tournament to annual incidence as it overestimates the extent of the injury situation.^3^ Tournament incidence was calculated per 100 players per tournament.
2. Match injuries included all new and recurring TL injuries that occurred solely during ‘The Hundred’ matches.^6^ Match incidence was calculated per 1000 days of play^4 6^ and 1000 player days.^2 3^ Match batting and bowling incidence were calculated per 10 000 deliveries faced and 10 000 deliveries bowled, respectively.^6^

### Data analysis

The number of players was taken from formal registration, and the number of balls bowled was taken from publicly available scorecards. Tournament injury prevalence was calculated by dividing the number of missed tournament days by the number of tournament days multiplied by the number of registered players, expressed as the percentage of players unavailable on any given day of the tournament. Any matches abandoned with no balls bowled were excluded from the number of matches played. The number of players and players’ ages were calculated as mean±SD. Injury data were presented as count, incidence and prevalence, with incidence and prevalence summarised as mean and 95% CI using the Poisson and Exact Binomial methods, respectively.^7^ Significant differences within and between the men’s and women’s injury incidence and prevalence values were identified if the 95% CI did not overlap.

### Patient and public involvement

Players and/or the public were not involved in this research’s design, conduct, reporting or dissemination.

### Equality, diversity and inclusion statement

The author team includes three women and seven men (the first author is a woman), comprises senior, mid-career and early-career investigators and has a variety of backgrounds, including academic researchers and practising sports medicine doctors and physiotherapists. The study population includes both women and men cricketers, with a spectrum of ages and playing experience. The influence of the gendered environment on injury is explored in the discussion.

## Results

On average, 149 (±10) men and 130 (±9) women players were registered for each tournament, with an average age of 28.5 (±4.8) and 25.1 (±4.8) years, respectively. Table 1 reports the exposures for each competition. A total of 164 men’s (45 TL; 119 NTL) and 127 women’s (31 TL; 96 NTL) injuries were recorded in 94 men and 77 women.

**Table 1:** Exposures from the 2021–2023 ‘The Hundred’

Exposure	2021		2022		2023	
	Men	Women	Men	Women	Men	Women
No of matches*	33	32	34	26	33	30
No of registered players	144	126	161	141	143	124
Tournament days	31	32	32	24	27	27
No. match balls bowled	5941	6085	6537	5004	5932	5646

*Number of matches played (abandoned matches excluded).

### Tournament incidence and prevalence

A similar overall injury incidence for the men’s and women’s competition was observed (men’s: 36.6, women’s: 32.5/100 players/tournament). In the men’s and women’s competition, NTL incidence (26.6 and 24.6/100 players/tournament, respectively) was higher than TL incidence (10.0 and 7.9/100 players/tournament, respectively). Injury prevalence in the men’s competition was 2.9% and 3.6% in the women’s competition. The three competitions had no differences in injury incidence and prevalence (table 2).

**Table 2:** All injuries, TL and NTL tournament incidence (100 players/tournament) and tournament prevalence (percentage of players unavailable on any given day of the tournament) with 95% CI

	2021	2022	2023	Overall
Men’s				
Incidence	36.1 (27.5 to 47.4)	34.8 (26.8 to 45.2)	39.2 (30.1 to 50.9)	36.6 (31.4 to 42.7)
TL incidence	13.2 (8.4 to 20.7)	7.5 (4.2 to 13.1)	9.8 (5.8 to 16.5)	10.0 (7.5 to 13.5)
NTL incidence	22.9 (16.3 to 32.2)	27.3 (20.3 to 36.7)	29.4 (21.7 to 39.7)	26.6 (22.2 to 31.8)
Prevalence	3.7% (1.1% to 7.8%)	2.2% (0.7% to 6.1%)	2.8% (0.7% to 6.8%)	2.9% (1.5% to 4.8%)
Women’s				
Incidence	39.7 (30.1 to 52.4)	29.8 (22.0 to 53.7)	28.2 (20.3 to 47.7)	32.5 (27.3 to 38.7)
TL incidence	7.1 (3.7 to 13.7)	8.5 (4.8 to 15.0)	8.1 (4.3 to 15.0)	7.9 (5.6 to 11.3)
NTL incidence	32.5 (24.0 to 44.2)	21.3 (14.9 to 30.4)	20.2 (13.6 to 29.8)	24.6 (20.1 to 30.0)
Prevalence	2.9% (0.8% to 7.7%)	4.2% (1.5% to 8.7%)	3.9% (1.3% to 8.8%)	3.6% (1.9% to 5.7%)

NTL, non-TL; TL, time-loss.

### Injury event

**Figure 1:**
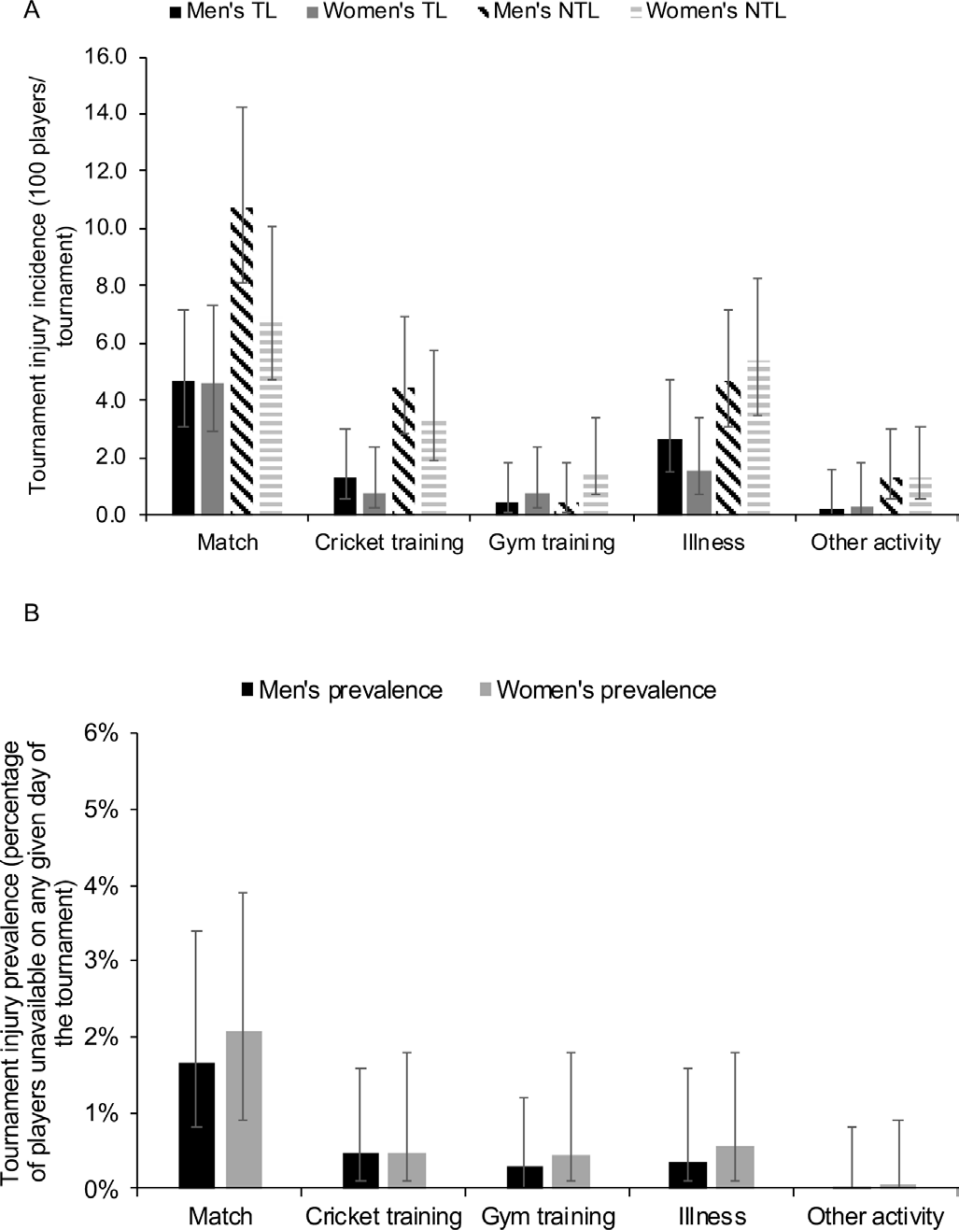
(A) The tournament time-loss (TL) and non-TL (NTL) injury incidence (100 players/tournament) and (B) the tournament injury prevalence (percentage of players unavailable on any given day of the tournament) by the injury event for the men’s and women’s competition, with 95% CI.

In the men’s competition, match TL (4.7/100 players/tournament) and NTL (10.7/100 players/tournament) incidence was higher than cricket training TL (1.3/100 players/tournament) and NTL (4.5/100 players/tournament), gym training TL (0.4/100 players/tournament) and NTL (0.4/100 players/tournament) and other activity TL (0.2/100 players/tournament) and NTL (1.3/100 players/tournament) incidence. Comparatively in the women’s competition, match TL (4.6/100 players/tournament) and NTL (6.9/100 players/tournament) incidence was higher than cricket training TL (0.8/100 players/tournament), gym training TL (0.8/100 players/tournament) and NTL (1.5/100 players/tournament) and other activity TL (0.3/100 players/tournament) and NTL (1.3/100 players/tournament) incidence. A similar rate of TL (men’s: 2.7, women’s: 1.5/100 players/tournament) and NTL (men’s: 4.7, women’s: 5.4/100 players/tournament) illness incidence was observed between both competitions (figure 1A). Tournament prevalence was highest in matches in the men’s and women’s competitions at 1.7% and 2.1%, respectively, but not higher than any other injury event (figure 1B). There were no differences in injury event incidence and prevalence between the men’s and women’s competition.

### Activity at the time of injury

Match fielding in the men’s and women’s competitions was the most common TL and NTL incidence. In the men’s competition, match fielding TL incidence (2.2/100 players/tournament) was higher than training fielding TL incidence. Whereas in the women’s competition, match fielding TL incidence (2.8/100 players/tournament) was higher than training bowling, with match fielding NTL injury incidence (3.8/100 players/tournament) higher than match bowling and training batting (table 3). Match fielding was the activity associated with the highest injury prevalence in both competitions (men’s: 0.7%, women’s: 1.4%) but not higher than any other activity.

**Table 3:** The number of injuries, tournament incidence (100 players/tournament) and prevalence (percentage of players unavailable on any given day of the tournament) by activity at the time of injury for the men’s and women’s ‘The Hundred’ competitions, with 95% CI

Activity	Men’s				Women’s			
	n	TL incidence	NTL incidence	Prevalence	n	TL incidence	NTL incidence	Prevalence
Fielding—match	27	2.2 (1.2 to 4.1)	3.8 (2.4 to 6.1)	0.7% (0.1% to 1.9%)	26	2.8 (1.6 to 5.1)	3.8 (2.3 to 6.4)	1.4% (0.4% to 2.9%)
Batting—match	21	0.9 (0.3 to 2.4)	3.8 (2.4 to 6.1)	0.3% (0.0% to 1.2%)	9	0.8 (0.2 to 2.4)	1.5 (0.7 to 3.4)	0.4% (0.1% to 1.8%)
Fielding—training	8	0.0 (0.0 to 0.0)	1.8 (0.9 to 3.6)	0.0% (0.0% to 0.8%)	8	0.5 (0.1 to 2.0)	1.5 (0.7 to 3.4)	0.3% (0.0% to 1.4%)
Bowling—training	10	0.9 (0.3 to 2.4)	1.3 (0.6 to 3.0)	0.4% (0.1% to 1.6%)	6	0.0 (0.0 to 0.0)	1.5 (0.7 to 3.4)	0.0% (0.0% to 0.9%)
Bowling—match	11	1.1 (0.5 to 2.7)	1.3 (0.6 to 3.0)	0.5% (0.1% to 1.6%)	4	0.8 (0.2 to 2.4)	0.3 (0.0 to 1.8)	0.2% (0.0% to 1.4%)
Batting—training	8	0.4 (0.1 to 1.8)	1.3 (0.6 to 3.0)	0.0% (0.0% to 0.8%)	2	0.3 (0.0 to 1.8)	0.3 (0.0 to 1.8)	0.2% (0.0% to 1.4%)

NB: no wicket-keeping injuries were recorded.

NTL, non-TL; TL, time-loss.

### Body region and location

The lower limb was the body region with the highest TL incidence (men’s: 3.3, women’s: 3.3/100 players/tournament). In both competitions, TL lower limb incidence was higher than the head and neck and the trunk region in the women’s competition (table 4). The upper limb body region was the highest NTL incidence in the men’s (8.0/100 players/tournament) and women’s (7.7/100 players/tournament) competition and was higher than the head and neck NTL incidence. Upper limb NTL incidence was higher than TL upper limb incidence in both competitions. Lower limb injuries were associated with the highest injury prevalence (men’s: 1.1%, women’s: 1.6%) but not higher than any other body region. In the men’s competition, thigh injuries were the most common TL body location injured (2.0/100 players/tournament). In contrast, the hand was the most common TL body region injured in the women’s competition (1.5/100 players/tournament). Hand injuries were the most common NTL injury in both competitions (men’s: 3.6, women’s: 3.3/100 players/tournament).

**Table 4:** The number of injuries, tournament injury incidences (100 players/tournament) and tournament injury prevalence (percentage of players unavailable on any given day of the tournament) with 95% CI by body regions and locations for the men’s and women’s ‘The Hundred’ 2021–2023

	Time-loss		Non-time-loss		Injury prevalence
	No injuries	Incidence	No injuries	Incidence	
*Men’s*					
** Head and neck**	**1**	**0.2 (0.0 to 1.6)**	**9**	**2.0 (1.0 to 3.9)**	**0.0% (0.0% to 0.8%)**
Head	*0*	*0.0 (0.0 to 0.0*)	*7*	*1.6 (0.7 to 3.3*)	*0.0% (0.0% to 0.8%*)
Neck	*1*	*0.2 (0.0 to 1.6*)	*2*	*0.4 (0.1 to 1.8*)	*0.0% (0.0% to 0.8%*)
**Upper limb**	**7**	**1.6 (0.7 to 3.3**)	**36**	**8.0 (5.8 to 11.1**)	**0.6% (0.1% to 1.9%**)
Shoulder	*2*	*0.4 (0.1 to 1.8*)	*11*	*2.5 (1.4 to 4.4*)	*0.1% (0.0% to 0.8%*)
Elbow	*1*	*0.2 (0.0 to 1.6*)	*5*	*1.1 (0.5 to 2.7*)	*0.0% (0.0% to 0.8%*)
Hand	*4*	*0.9 (0.3 to 2.4*)	*16*	*3.6 (2.2 to 5.8*)	*0.5% (0.1% to 1.6%*)
Wrist	*0*	*0.0 (0.0 to 0.0*)	*3*	*0.7 (0.2 to 2.1*)	*0.0% (0.0% to 0.8%*)
** Trunk**	**10**	**2.2 (1.2 to 4.1**)	**20**	**4.5 (2.9 to 6.9**)	**0.7% (0.1% to 1.9%**)
Chest	*0*	*0.0 (0.0 to 0.0*)	*2*	*0.4 (0.1 to 1.8*)	*0.0% (0.0% to 0.8%*)
Abdomen	*5*	*1.1 (0.5 to 2.7*)	*3*	*0.7 (0.2 to 2.1*)	*0.4% (0.1% to 1.6%*)
Lumbar spine	*5*	*1.1 (0.5 to 2.7*)	*12*	*2.7 (1.5 to 4.7*)	*0.3% (0.0% to 1.2%*)
Thoracic spine	*0*	*0.0 (0.0 to 0.0*)	*3*	*0.7 (0.2 to 2.1*)	*0.0% (0.0% to 0.8%*)
**Lower limb**	**15**	**3.3 (2.0 to 5.6**)	**33**	**7.4 (5.2 to 10.4**)	**1.1% (0.4% to 2.6%**)
Buttocks and pelvis	*0*	*0.0 (0.0 to 0.0*)	*1*	*0.2 (0.0 to 1.6*)	*0.0% (0.0% to 0.8%*)
Hip and groin	*1*	*0.2 (0.0 to 1.6*)	*5*	*1.1 (0.5 to 2.7*)	*0.0% (0.0% to 0.8%*)
Thigh	*9*	*2.0 (1.0 to 3.9*)	*10*	*2.2 (1.2 to 4.1*)	*0.6% (0.1% to 1.9%*)
Knee	*2*	*0.4 (0.1 to 1.8*)	*7*	*1.6 (0.7 to 3.3*)	*0.1% (0.0% to 0.8%*)
Lower leg	*2*	*0.4 (0.1 to 1.8*)	*0*	*0.0 (0.0 to 0.0*)	*0.3% (0.0% to 1.2%*)
Ankle	*0*	*0.0 (0.0 to 0.0*)	*6*	*1.3 (0.6 to 3.0*)	*0.0% (0.0% to 0.8%*)
Foot	*1*	*0.2 (0.0 to 1.6*)	*4*	*0.9 (0.3 to 2.4*)	*0.1% (0.0% to 0.8%*)
*Women’s*					
**Head and neck**	**2**	**0.5 (0.1 to 2.0**)	**6**	**1.5 (0.7 to 3.4**)	**0.2% (0.0% to 1.4%**)
Head	*2*	*0.5 (0.1 to 2.0*)	*5*	*1.3 (0.5 to 3.1*)	*0.2% (0.0% to 1.4%*)
Neck	*0*	*0.0 (0.0 to 0.0*)	*1*	*0.3 (0.0 to 1.8*)	*0.0% (0.0% to 0.9%*)
**Upper limb**	**9**	**2.3 (1.2 to 4.4**)	**30**	**7.7 (5.4 to 11.0**)	**1.2% (0.4% to 2.9%**)
Shoulder	*3*	*0.8 (0.2 to 2.4*)	*12*	*3.1 (1.7 to 5.4*)	*0.4% (0.1% to 1.8%*)
Elbow	*0*	*0.0 (0.0 to 0.0*)	*4*	*1.0 (0.4 to 2.7*)	*0.0% (0.0% to 0.9%*)
Hand	*6*	*1.5 (0.7 to 3.4*)	*13*	*3.3 (1.9 to 5.7*)	*0.8% (0.2% to 2.2%*)
Wrist	*0*	*0.0 (0.0 to 0.0*)	*1*	*0.3 (0.1 to 1.8*)	*0.0% (0.0% to 0.9%*)
**Trunk**	**1**	**0.3 (0.0 to 1.8**)	**12**	**3.1 (1.7 to 5.4**)	**0.0% (0.0% to 0.9%**)
Chest	*0*	*0.0 (0.0 to 0.0*)	*1*	*0.3 (0.0 to 1.8*)	*0.0% (0.0% to 0.9%*)
Abdomen	*0*	*0.0 (0.0 to 0.0*)	*4*	*1.0 (0.4 to 2.7*)	*0.0% (0.0% to 0.9%*)
Lumbar spine	*1*	*0.3 (0.0 to 1.8*)	*5*	*1.3 (0.5 to 3.1*)	*0.0% (0.0% to 0.9%*)
Thoracic spine	*0*	*0.0 (0.0 to 0.0*)	*2*	*0.5 (0.1 to 2.0*)	*0.0% (0.0% to 0.9%*)
**Lower limb**	**13**	**3.3 (1.9 to 5.7**)	**27**	**6.9 (4.7 to 10.1**)	**1.6% (0.6% to 3.3%**)
Buttocks and pelvis	*0*	*0.0 (0.0 to 0.0*)	*1*	*0.3 (0.0 to 1.8*)	*0.0% (0.0% to 0.9%*)
Hip and groin	*0*	*0.0 (0.0 to 0.0*)	*2*	*0.5 (0.1 to 2.0*)	*0.0% (0.0% to 0.9%*)
Thigh	*5*	*1.3 (0.5 to 3.1*)	*9*	*2.3 (1.2 to 4.4*)	*0.7% (0.2% to 2.2%*)
Knee	*2*	*0.5 (0.1 to 2.0*)	*6*	*1.5 (0.7 to 3.4*)	*0.4% (0.1% to 1.8%*)
Lower leg	*4*	*1.0 (0.4 to 2.7*)	*2*	*0.5 (0.1 to 2.0*)	*0.5% (0.1% to 1.8%*)
Ankle	*2*	*0.5 (0.1 to 2.0*)	*3*	*0.8 (0.2 to 2.4*)	*0.1% (0.0% to 0.9%*)
Foot	*0*	*0.0 (0.0 to 0.0*)	*4*	*1.0 (0.4 to 2.7*)	*0.0% (0.0% to 0.9%*)

Bold values indicate body regions, non-bold values indicate body locations.

### Match incidence and prevalence

The match incidence in the men’s competition was 1.6/1000 player days and 233.3/1000 days of play. In the women’s competition, match incidence was 1.7/1000 player days and 216.9/1000 days of play. No differences were observed in match incidences between the men’s and women’s competitions (table 5).

**Table 5:** The average match injury incidence rates for men’s and women’s ‘The Hundred’ competition, with 95% CI

	Men’s	Women’s
Match injuries/1000 player days	1.6 (1.0 to 2.4)	1.7 (1.1 to 2.7)
Match injuries/1000 days of play	233.3 (152.1 to 357.9)	216.9 (136.6 to 344.2)
Bowling match injuries/10 000 deliveries bowled	2.7 (1.1 to 6.5)	1.8 (0.6 to 5.6)
Batting match injuries/10 000 balls faced	2.2 (0.7 to 6.7)	1.8 (0.6 to 5.6)

## Discussion

This is the first study to explore the injury rates and types during a novel format of men’s and women’s franchise cricket in England and Wales. A similar tournament and match injury incidence was observed between the men’s and women’s competitions. Fielding during matches was associated with the highest injury incidence. Lower limb injuries presented the highest TL incidents, with upper limb injuries being the most frequent NTL incidents.

### Injury incidence

A comparison of elite men’s and women’s injury rates in Australian cricket showed a similar incidence of medical attention injuries, as found in this study.^5^ However, a higher rate of injury in domestic T20 and one-day men’s cricket was observed compared with women’s domestic T20 and one-day cricket.^5^ In domestic cricket, men play more matches than women and have more consecutive game days due to multiday format fixtures. As a result, men players are exposed to more cricket with reduced recovery periods, which may increase susceptibility to injury compared with women cricketers.^5^ However, no difference was observed in ‘The Hundred’, a competition with similar match scheduling and rest days between the men’s and women’s competitions. ‘The Hundred’ men’s and women’s teams also had access to the same medical staff and resources, which has been thought to influence recorded injury rates.^8^ These factors may explain the similarity in the tournament and match incidences observed.

Although direct comparison of injury rates is not always possible, match injury incidence in the men’s ‘The Hundred’ was similar to one-day domestic men’s cricket (254/1000 days of play (95% CI 231 to 280)) but higher than men’s domestic T20 (136/1000 days of play (95% CI 121 to 152)) in England and Wales.^3^ Match bowling injuries occurred at a similar rate observed in Australian men’s T20 (1.5/1000 overs bowled) and one-day cricket (2.2/1000 overs bowled).^9^ Compared with the international one-day men’s World Cup, match bowling and match batting injuries occurred at a similar rate to ‘The Hundred’ (2.7/1000 overs bowled and 2.2/10 000 balls faced, respectively).^10^ Considering the inconsistencies and scarcity of data available in women’s cricket, it is beneficial to make comparisons to men’s cricket to understand the level of risk in the women’s ‘The Hundred’ compared with other cricket formats. The women’s ‘The Hundred’ match injury incidence is comparable to men’s domestic T20 (136 injuries/1000 days of play (95% CI 121 to 152)) and one-day cricket (254 injuries/1000 days of play (95% CI 231 to 280)) in England and Wales.^3^ Ongoing ‘The Hundred’ injury surveillance is required to confirm these findings.

NTL incidence was higher than TL incidence in both competitions. This has been observed previously in men’s and women’s cricket tournaments, where NTL incidence was reported between 3.8 and 5.8 times higher than TL incidence.^10 11^ In tournaments, players are more likely to play with injuries that may result in TL outside of a competition. Pressure from coaches to play and on medical staff may see players return to matches quicker and influence NTL incidence during tournaments.^11^ There is financial incentive to play in ‘The Hundred’ so players may not report injury prior to or under-report in competition. In turn, this could have extenuated the injury problem. The effect of players competing in ‘The Hundred’ with injuries could impact player availability for the remainder of the domestic cricket season, as players’ income in the remaining domestic season is not affected by availability. Future work should explore this to understand the full impact of ‘The Hundred’.

### Injury prevalence

On any given day of ‘The Hundred’, 2.9% of men and 3.6% of women were unavailable to train or play. This prevalence rate is lower than observed in domestic men’s cricket, between 7.5% and 12.5%,^2–4^ and similar to domestic women’s cricket in England and Wales at 4.1%.^12^ Interestingly, injury prevalence has been reported between 4.6% and 5.1% in other cricket tournaments,^10 11^ closer to the observed rate in this study. The lower prevalence rate in tournament cricket could be related to the higher NTL incidence, which does not contribute to injury prevalence rates. The financial incentive to play in ‘The Hundred’ may have contributed to the lower injury prevalence recorded, encouraging players to return from injury sooner. Furthermore, players injured before ‘The Hundred’ with an expected return date close to the end of the tournament are likely to withdraw from the competition, whereas, in domestic cricket, these players will remain with the club influencing the prevalence rates. This is especially relevant for lumbar spine stress fractures, which typically require four to six months recovery.^13 14^ The full prevalence would not be captured in ‘The Hundred’ injury surveillance but would be in year-round injury surveillance. Bowlers’ workload is also limited to 20 deliveries per game (3.2 overs) in ‘The Hundred’ which is less than all other formats. This reduces exposure for bowlers, who have been reported as the players with the highest injury prevalence.^5^

### Activity at injury and injury location

In both competitions, match fielding was associated with the highest TL and NTL incidence. Fielding has been reported as the activity with the highest TL incidence in men’s and women’s T20.^3 11^ In men’s T20 cricket, it is a new finding that fielding presents a greater risk of injury than bowling.^3^ This warrants future studies to include format-specific injury profiles to validate the injury risk that short-formats may present. Limited-overs formats (T20 and one-day) are more demanding in men’s cricket, with all players required to sprint more than in multiday cricket.^15 16^ The greater sprinting and the association between high-speed running and hamstring injury risk^17^ can expose players to increased injury risk. Coincidently, thigh injuries were the highest TL incidence in the men’s ‘The Hundred’ and a higher hamstring incidence rate in limited-overs cricket has been observed compared with multiday cricket.^18^ Due to the increased demands and exposure to limited-overs cricket since the introduction of ‘The Hundred’, players may have a heightened risk of sustaining a hamstring injury. Future research should aim to understand the demands of ‘The Hundred’, which will have practical implications for medical and conditioning staff in preparing players appropriately. The most common NTL injury in the men’s competition was the hand, often associated with fielding in men’s cricket.^19^ Improvements in fielding practices may reduce the risk of hand injuries in the men’s ‘The Hundred’.

Fielding is associated with the highest injury rate in all formats in women’s cricket,^12 20^ with catching and throwing the specific activities having the highest incidence.^11^ In this study, hand injuries were the highest TL and NTL incidence, consistent with previous research in elite women’s cricket.^12 20^ Hand injuries are often attributed to fielding (and mis-fielding), specifically when catching a ball.^20^ Shoulder injuries were the second highest NTL incidence in the women’s competition, which have been associated with throwing^11^ and can impact primary position.^21 22^ In limited-overs cricket, as exclusively played in women’s domestic cricket, fielding is highly important to minimise the runs scored by the opposition. As a result, players may dive to prevent the ball from reaching the boundary and perform more throws at increased speeds. This may increase the risk of developing a shoulder injury as an increased throwing workload has been identified as a risk factor for upper limb injuries.^23^ Improving fielding practices and workload management, may reduce the risk of fielding, hand and shoulder injuries in women’s ‘The Hundred’.

### Limitations

Numerous medical staff from each team were recording injuries, which may have led to some inconsistencies. Mitigation measures were implemented, including the lead author checking all injury data and consulting with the gatekeeper when needed and the ECB mandating standards for injury records. Nevertheless, there is a small risk of data inaccuracy. It is possible players may not have reported injuries sustained elsewhere until competing in ‘The Hundred’ to receive the financial benefit. Similarly, the workload of players from other formats could have influenced injury risk, resulting in both these factors leading to an overestimation of the injury incidence. Only injuries sustained in ‘The Hundred’ were included in the study, as such players with injuries entering the tournament were not captured. Although 3 years of data are included, additional data from future years are required to confirm these findings and to monitor any injury prevention initiatives that are implemented. It should be noted that the number of matches in the women’s 2022 competition was reduced due to a clash with the Commonwealth Games, meaning the match schedule was not the same as the men’s competition, which could have influenced injury incidence.

## Conclusion

A similar injury profile was observed between the men’s and women’s ‘The Hundred’; match fielding presented the greatest injury risk, with lower limb TL injuries and upper limb NTL injuries the most frequent. ‘The Hundred’ men’s appears to present a greater risk of injury than T20, although similar to one-day cricket, with the women’s ‘The Hundred’ presenting a similar risk to both one-day and T20 cricket. Additional ‘The Hundred’ data are required to confirm these findings. This study begins to provide evidence of the extent of the injury problem in ‘The Hundred’, allowing future studies to identify risk factors and develop prevention strategies, ultimately improving player welfare.

## Data Availability

All data relevant to the study are included in the article or uploaded as online supplemental information. Not applicable.
